# Supplementary material for: Copper to Tuscany – Coals to Newcastle? The dynamics of metalwork exchange in early Italy
Source: PLoS One. 2020 Jan 22;15(1):e0227259. doi: 10.1371/journal.pone.0227259 (PMC6975538; doi:10.1371/journal.pone.0227259)
Supplement: Attachment — Submitted filename: Dolfini-et-al-Response_to_reviewers.docx [file pone.0227259.s001.docx]

**Dolfini A, Angelini, I & Artioli, G’s response to reviewers**

Dear Professor Biehl,

We are delighted that PloS One has expressed interest in publishing a revised version of the manuscript titled: ‘Copper to Tuscany – Coals to Newcastle? The dynamics of metalwork exchange in early Italy’. We are extremely grateful for the helpful comments provided by the journal editors and anonymous reviewers, as they have enabled us to enhance the manuscript significantly. In a few instances, however, we have exercised our informed judgement not to accept them, or to accept them partly. Our decisions, and the criteria informing them, are detailed below.

**Editorial comments**

**Comment**: In your manuscript, please provide additional information regarding the specimens used in your study. Ensure that you have reported specimen numbers and complete repository information, including museum name and geographic location. If permits were required, please ensure that you have provided details for all permits that were obtained, including the full name of the issuing authority etc.

**Response**: We have implemented all recommendations: see revised manuscript and Table 2.

**Comment**: We note that [Figure(s) 1-6] in your submission contain [map/satellite] images which may be copyrighted etc.

**Response**: Please note that this is partly incorrect. The only maps attached to the manuscript are Figs 1, 5 and 6. Fig. 1 has now been redrawn using a CC BY 4.0 base map from the US Geological Survey. Fig 5 has been turned into an inset of Fig 1 following Reviewer #3’s recommendation. Figs 2 and 4 are original images drawn and supplied by sympathetic colleagues, who have consented to their publication within this article; the authors are acknowledged in the captions and thanked in the Acknowledgements, but the images themselves are not copyrighted. Figs 3 and 6 [now 5] are being reproduced by informed consent of copyright holders: see content permission forms attached. Both Rivista di Scienze Preistoriche and Rivista Mineralogica Italiana have confirmed that the copyrights of the two images lie with the authors. The image captions have been modified following the editor’s suggestions.

**Reviewer’s #1 comments**

**Comment 1**: The title of the article is different in the MS submission page and in the main body text. Make sure that the MS title is consistent.

**Response**: This has now been corrected.

**Comment 2**: Lines 63-64: the authors document the ternary alloy of Cu-As-Sb alloy as ‘solely’ in central Italy etc.

**Response**: Thank you for pointing this out; passage corrected.

**Comment 3**: For Table 1, please give both 1σ and 2σ probabilities of C14 dates.

**Response**: Comment ignored as Table 1 has been pulled out of the manuscript following Reviewer #3’s recommendations (see below).

**Comment 4**: Line 142 & 738: remove the word ‘negative’ as it does not strengthen the expression in the sentence etc.

**Response**: Point taken – ‘negative’ removed and passages corrected.

**Comment 5**: For the chronologies argued between lines 249-263, a recent EU funding is mentioned and a paper presentation in the upcoming EAA is cited etc.

**Response**: Point partly taken. All reviewers comment about the excessive weight given to the chronology of these objects. On reflection, we agree with them. We have comprehensively revised all passages discussing the chronology of the objects, doing two things: (1) drawing attention to published works that support our chronological proposals, where appropriate; and (2) toning down the chronological discussion (which is important, but not central to the argument), especially in the case of speculative or unconfirmed dating proposals, such as the object supposedly made of French copper.

**Comment 6**: For the objects with suggested provenance from outside Tuscany, antimony and arsenic bearing cupriferous ores are preferred also on the grounds of close proximity. The authors should clarify a) why they have completely ruled out co-smelting of copper ores and arsenic/antimony ores? b) As traces of Sb-containing minerals were detected, and considering the polymetallic nature of the region, could there be another undetected source of Sb available?

**Response**: Point partly taken; co-smelting briefly mentioned in the revised script. However, the point they make is not central to the argument; what we discuss in this passage is not the nature of the ore in the smelting charge but whether it comes from Tuscany or not. The bottom line here is that it is normally impossible to prove or disprove co-smelting of mixed ores by looking at the LI signatures of finished objects. Dwelling on this problem would take the argument off track.

**Comment**: The assertions in the paragraph (lines 586-596) ground on far too many unconfirmed chronology along with thin artefactual evidence (an axe head) etc.

**Response**: Point taken and chronological inference toned down (see response to comment 5).

**Reviewer’s #2 comments**

**Comment 1**: Line 522: there is a mistake: it should be '... high As, high Sb' not 'As'

**Response**: Well spotted! Typo corrected.

**Comment 2**: Lines 694-696: it is surprising that Slovakian Ore Mountains are not mentioned as possible source of copper for the 'central and eastern European communities'

**Response**: Thank you for spotting the omission; passage amended accordingly.

**Comment 3**: Lines 393-398: The database for ore deposits in Italy sounds very impressive. The OXALID is open access, are there plans to make the database of the Padova University also an open access resource?

**Response**: Most of the Tuscan data have been retrieved from published geological literature and available PhD dissertations. Our data complementing the literature are of course available on request.

**Reviewer’s #3 comments**

**Major comment 1**: Most of the objects included in this study came from unsecured locations and do not have precise archaeological context. Their dating to the Chalcolithic period is based on “a recent EU-funded project aiming to date early Italian metals, including those discussed here, by combined morphometric analysis, radiocarbon dating, and a critical review of find contexts [71].” It is important to note; however, that reference #71 is a recent conference abstract. If we accept the author’s claim that “as is typical of Copper Age Italy, they are mostly stray finds, suggesting intentional deposition in the open landscape” the paper can be published. Maybe I am too conservative, but I am used to analyze only archaeological objects that come from a secure, well-constrained archaeological context. I think that the editor should decide whether the use of these objects warrants the publication of the paper.

**Response**: We thank the reviewer for raising this important issue. In fact, their comments raise two separate issues, which we address separately here: (1) unconfirmed provenance of the objects and (2) object chronology.

Issue (1): it is true that most axe-heads in our sample are stray finds. As argued in the article, this is a function of Copper Age deposition practices, whereby axe-heads were often deposited in the open landscape as single finds. They were mostly discovered during farming work and other non-archaeological activities and often entered museum collections as donations or purchases (Dolfini 2008). Although we agree with the reviewer that this is not ideal, the find spots or areas these objects are from are known. This is sufficient to utilise them for the provenance exercise the research is concerned with. Unfortunately, comparatively few early metals from central Italy are from well-excavated archaeological sites. When the context is known, interpretations can often be more in-depth (see the case of Pianizzoli discussed in the paper). Yet we have to work with the archaeology we have, not the archaeology we wish we had. A recent trend in archaeological science (and archaeometallurgy in particular: e.g. Pollard 2018) sees old collections being given a new lease of life through the application of recently developed scientific methods. Our research is part of this growing trend. We are proud to be able to show that so much new data concerning provenance, exchange, recycling, and circulation can be extracted from old museum collections. This, we argue, is a strength of the work, not a weakness.

Issue (2): the comment is partly due to a misunderstanding. The *overall* chronology of the objects in our sample is undisputed. All objects are all securely dated to the Italian Copper Age (c.3600-2200 BC) through multiple, and often co-occurring, markers including typology, chemical composition and, where available, object associations and radiocarbon dating. This is discussed in published works cited in the article (e.g. De Marinis 2006; Dolfini 2010). What the recent project mentioned above was concerned with, is the *detailed* chronology of the objects, which – we recognise – may still be open to debate in certain circumstances. Following this and other reviewers’ advice, we have comprehensively revised the chronological discussion, clarifying that all objects are securely dated to the Copper Age (and adding references to published work where necessary) and toning down the discussion of the detailed object chronology when not fully supported by radiocarbon or contextual data. However, we still highlight when objects are securely, or probably, dating to the 4^th^ millennium BC, as this has significant implications for the interpretation of early copper sourcing and exchange (see Results and Discussion, and subsequent sections).

**Major comment #2**: The paper is too long and too convoluted. Once we accept the dating of the objects, there is one striking result that should be emphasized: 55-75% of metal items were produced from Tuscan copper, either from Southern Tuscany or from the Apuanian Alps, and 25-45% come from outside this region. This is a very important observation that merits the publication of this paper. The rest of the argumentations should be scaled down, and the use of ED models should be mentioned but not presented graphically. I will outline below which of the figures and tables can be omitted, and also comment on other minor aspects of the paper.

**Response**: We respectfully disagree with Reviewer #3’s comment that ‘the paper is too long and convoluted’, noting that neither the other reviewers nor the editors share their concerns. The reviewer seems to suggest that the paper should be turned into a single-issue article solely concerned with the (admittedly important) finding that ¼ of Copper Age objects in our sample were not made from Tuscan copper. However important, this is a discovery that, in and of itself, may beg the oft-asked ‘so what’ question. The real significance of the paper (and the reason we have submitted it to PloS One, not some second-rank journal) lies with putting this result in context. Learning that early central Italian metals were part of a broader procurement and exchange circuit that stretched all over the mid/upper Tyrrhenian coast, the Western Alps, and southern France; that this circuit was in operation at the same time as other circuits stretching into the northern Alps and the Balkans; and that these circuits were largely mutually exclusive; all this gives significance to the data and paints a bold new picture of the dynamics of metal exchange in pre-Bronze Age Europe. Importantly, our research results are independently confirmed, and thus strengthened, by research carried out on the early Alps with a different approach and method, as discussed in the paper. These, we strongly argue, are strengths, not weaknesses, of our paper. Having said so, we acknowledge that the two major research results (i.e. alien copper in early Tuscany and super-regional sourcing and exchange circuits) were not sufficiently highlighted in the original paper. We have thus redrafted parts of the last two sections, and the Conclusion especially so. In the latter, we have now brought home the two main research results and have toned down all other results following the reviewer’s helpful recommendations. However, we have not taken on board many of their minor comments pushing towards a much shorter single-issue article, as detailed below. Note that all line numbers refer to the original submission.

**Reviewer #3’s minor comments**

**Comment 1**: The abstract should be more informative, and should include real data.

**Response**: We are puzzled by this comment. On reading the abstract carefully, we find it informative (scope, aim and main results of the research are duly outlined) and supported by real data (notwithstanding the lack of detail typical of abstracts). However, if the editors wish to put forwards specific suggestions for improving the abstract, we will consider them.

**Comment 2**: Figure 1 should include a few contemporary cities so readers who are unfortunate to be non-Italian will have an easy way to put the finds in geographical context. Figure 5 should be an insert in Figure 1.

**Response**: Thank you for pointing this out. Major Italian cities now added to the map and Fig. 5 turned into an inset.

**Comment 3**: Figures 2 and 3 and Tables 1 and 2 are not needed.

**Response**: Table 1 has been pulled out and all other tables renumbered. We disagree about the rest. Table 2 is a synopsis of the chronology discussed in the paper. We believe it provides a helpful aid for readers not familiar with Italian prehistory (arguably, the point above about geography also applies to chronology). The same goes for Figs 2 and 3; they illustrate the archaeological introduction of Copper Age Italy lying the foundations for the research. However, if the editors feel strongly about removing Figs 2-3, we will.

**Comment 4**: Line 112: Define LIA here

**Response**: The acronym is defined where it is first introduced, i.e. line 92. The acronym is consistently used thereafter.

**Comment 5**: The meaning “soapstone” for steatite should appear already in line 127.

**Response**: Point taken and passage amended accordingly.

**Comment 6**: Figure 6: The map should be modified. Replace the words "unit" "province" w/ the rock type/s and geological periods (e.g., Jurassic limestone and dolomite, Tuscan ultramafic 50 Myr pluton/unit).

**Response**: We respectfully disagree with the reviewer. The map was taken from a peer-reviewed journal article; it uses the accepted geological terminology, which is also the terminology we use in our article. We have just translated the labels and keys into English. We believe the map is a useful aid to readers as it helps them locate the geological formations on the ground. However, if the editors feel strongly about this issue, it can be pulled out, noting that this would make the article harder to follow.

**Comment 7**: Lines 285 – 301 should be rewritten and included timing (in Myr) of the geological events, as this will be used later in the Pb-Pb plots (see below).

**Response**: Approximate time of formations are now reported in Table 4. However, because many deposits bear multiple formation and/or remobilization at different times, it is not straightforward to link a deposit’s age to each model.

**Comment 8**: Table 4: Should add age of formation to all ores in Myr.

**Response**: Geological settings were added for the formations, though it is hard to give absolute dates, since many of the original Jurassic ophiolites (150-160 My old) have been embedded into later sediments and turbidites by the Apennine orogeny, so the actual age of the formation is related to the age of the orogeny (Cretaceous and Tertiary), with even later metamorphic remobilization (as young as 15-20 My old) and ore formation from the fluids of the plutonic complex, forming the typical skarn deposits.

**Comment 9**: The phrasing “The latter two fields have a clear metamorphic and/or hydrothermal character resulting in substantially higher 207Pb/204Pb and 208Pb/204Pb values compared with the Ligurian-Apennine ophiolites, is not correct. There might be other reasons: younger age of formation or higher U and Th concentrations. This issue can be resolved w/ appropriate use of the literature

**Response**: We have now amended the phrase to make it clearer: “The latter two fields have a clear metamorphic character and younger age resulting in substantially higher ^207^Pb/^204^Pb and ^208^Pb/^204^Pb values compared with the Ligurian-Apennine ophiolites”.

**Comment 10**: Figure 7a, b, c should be modified. The authors should add Pb-Pb model age curves. Also, they should verify that there is no mass dependent fractionation (an instrumental artifact) in the database that they use.

**Response**: There is no mass dependent fractionation in our data (Alps, Tuscany, Western Alps). As a matter of fact, when the data have been modelled appropriately (**Nimis P., Omenetto P, Giunti I., Artioli G., Angelini I.**: Lead isotope systematics in hydrothermal sulphide deposits from the central-eastern Southalpine (northern Italy). Eur. J. Miner. **24**, 23-37, 2012. **DOI**: 10.1127/0935-1221/2012/0024-216; **Nimis P., Omenetto P., Stasi G., Canovaro C., Dal Sasso G., Artioli G., Angelini I.:** Lead isotopes systematics in ophiolite-associated sulphide deposits from the Western Alps and Northern Apennine (Italy). Eur. J. Mineral. **30**, 17-31, 2017. **DOI**: 10.1127/ejm/2018/0030-2696), they fit very well the Pb-Pb model curves and show no systematic instrumental biases. Of course, we cannot be totally certain that the literature data are without errors. In the figures we prefer maintaining the visual estimation of the curves as a guide to the eyes, rather than the more rigorous model curves.

**Comment 11**: Lines 383-384: They should report the measured 6/4, 7/4, 8/4 ratios of SRM981 and the errors on each one of them (2σ) and the number of independent runs during this study.

**Response**: The reported errors are the normal propagation through the runs.

**Comment 12**: The authors should emphasize more the issue of mixing, and using the modified Figure 7 they will be better equipped to address this issue.

**Response**: Mixing is a common process in Bronze Age metallurgy, but there is yet sparse evidence that it was practised on a significant scale prior to that, except for the remelting of individual objects into new ones (i.e. without mixing metals from different sources), which is highlighted by both our research and Bray’s research on early Britain (Bray 2012, reference 122 in our bibliography; see also see Villa, I.M., 2016, Provenancing bronze: exclusion, inclusion, uniqueness, and Occam’s razor. In: *Isotopic landscapes in bioarchaeology*, Springer, Berlin, Heidelberg, Pp. 141-154). The issue is touched upon in lines 508-515 (lines 534-542 in the revised manuscript) and discussed in lines 647-664 (lines 676-694 in the revised manuscript). We argue here that copper mixing probably starts in the 3^rd^ millennium BC on a relatively small scale, but the data may be interpreted differently. We therefore do not think it appropriate to emphasise the issue further.

**Comment 13**: “Fig 8. LI signatures of central Italian metal objects (labelled ‘Rinaldone’). 2D projections of the three-dimensional space defined by the measured isotopic ratios 206Pb/204Pb, 207Pb/204Pb, and 208Pb/204Pb.” I do not understand why they needed to label the object “Rinaldone”, and I do not see the 2D projection. All I see are two conventional Pb isotopic plots. In order to simplify this figure, I would downplay the symbols of the ores and add “fields” that mark their typical isotopic values. Also, Figure 8 should be part of the results.

**Response**: we have changed the labels and caption of fig 8 and subsequent figures from ‘Rinaldone’ to ‘central Italian objects’. The “conventional” Pb isotopic plots are actual 2D projections of the measured data in the 3D isotopic space. We prefer maintaining the available points because the simple outline of the fields do not give justice to the number of points defining the field and their density. For this, the KDE densities are much better, though superimposing several KDE fields would generate severe overlaps and confound the reader.

**Comment 14**: Figure 9 is not needed.

**Response**: We respectfully disagree. We make a point of using the KDE models, as they are not frequently used and they are a better graphical and statistical estimate of the ore fields in the isotopic space, as they represent the true density of points available to build each model. See also Pollard 2018 for the usefulness of KDE models.

**Comment 15**: Consider combining Tables 5 and 6.

**Response**: This is not practical. A combined table would be extremely large and would require considerable downsizing on publication, potentially making it hard to read.

**Comment 16**: Lines 508 – 509: In order to test for mixing try plotting 6/4 versus 1/[Pb].

**Response**: The mean concentration of [Pb] in the objects is not known at the moment, as the chemistry was only performed by point analyses using EPMA. The semi-quantitative presence of Pb and elements other than Cu can be assessed, but no reliable quantitative analysis is available.

**Comment 17**: Lines 519 – 520, this is part of the most important findings of this paper and should be presented much earlier.

**Response**: This comment is puzzling. This is the place where we discuss the data, and the data are discussed in their logical order, i.e. central/southern Tuscany first, then Apuanian Alps/mixed copper, and all other sources – Moving this earlier (where?) would confuse the argument.

**Comment 18**: Lines 571 – 574 are not needed in light of what is written in lines 578 – 579.

**Response**: Again, a baffling comment, which we have ignored. The two passages discuss two different datasets and interpretations. They are not similar and, in our view, should stay where they are and as they are.

**Comment 19**: Lines 644 – 645: This is too an important observation and should be mentioned much earlier in the paper, including in the abstract.

**Response**: This comment may be based on a misunderstanding. Compositional patterns by object type are not an original result of our research – this is something that has long been known, as the many references cited in line 645 duly clarify.

**Comment 20**: The use of the term “Oxford group’ method” is not clear, and when I looked at reference 121, the term became even more obscure to me.

**Response**: Thank you for raising the issue. The term has been changed into ‘Oxford system’, which is the accepted way to call the method (Pollard 2018). A brief explanation of the method, i.e. ‘charting changes in trace element composition and alloy type over time and geography’ has been added where the term is first introduced, and so has a reference to Pollard 2018, which provides a comprehensive presentation to the Oxford System. Giving further details about a method not employed in our research lies outside the paper’s scope.

**Comment 21**: Figure 10: Use different symbol types (e.g., dots versus stars) for objects and ores.

**Response**: Done; thank you for the observation.

**Comment 22**: Lines 680 – 708: Too long and too speculative. Make it substantially shorter.

**Response**: As detailed in our response to major comment 2 (above), we profoundly disagree with this recommendation. This is arguably one of the two most important results of the research. It is certainly not speculative; the discussion is grounded in published LI data (see references cited). We note that the other reviewers do not raise this issue.

**Comment 23**: Figure 11 is not needed.

**Response**: We respectfully disagree. We argue that it is important to show that the ore model of the Ligurian mines is reliable and well defined. Consequently, not finding copper from these ore deposits in our sample is not due to a sample or instrumental bias but it is a real datum.

**Comment 24**: Conclusions: should be rewritten emphasizing first the important messages of the paper.

**Response**: We thank the reviewer for this important observation. We have redrafted the conclusion to highlight the two major results of the research and tone down the others.

**Comment 25**: References: There are a few typos in the references.

**Response**: Thank you. References now fully checked and formatted.
